# Advances in the Use of Stem Cells in Veterinary Medicine: From Basic Research to Clinical Practice

**DOI:** 10.1155/2016/4516920

**Published:** 2016-06-09

**Authors:** Melissa Medeiros Markoski

**Affiliations:** Laboratório de Cardiologia Molecular e Celular, Fundação Universitária de Cardiologia/Instituto de Cardiologia, Princesa Isabel Avenue 370, 90620-001 Porto Alegre, RS, Brazil

## Abstract

Today, several veterinary diseases may be treated with the administration of stem cells. This is possible because these cells present a high therapeutic potential and may be injected as autologous or allogenic, freshly isolated, or previously cultured. The literature supports that the process is safe and brings considerable benefits to animal health. Knowledge about how adult stem cells modulate the molecular signals to activate cell homing has also been increasingly determined, evidencing the mechanisms which enable cells to repair and regenerate injured tissues. Preclinical studies were designed for many animal models and they have contributed to the translation to the human clinic. This review shows the most commonly used stem cell types, with emphasis on mesenchymal stem cells and their mechanistic potential to repair, as well as the experimental protocols, studied diseases, and species with the highest amount of studies and applications. The relationship between stem cell protocols utilized on clinics, molecular mechanisms, and the physiological responses may offer subsidies to new studies and therefore improve the therapeutic outcome for both humans and animals.

## 1. Introduction

In the last 20 years, considerable attention has been given to the research about the biology of stem cells. As a result, there was a significant increase in the understanding of its characteristics and, at the same time, the therapeutic potential for its application in different areas [1–4]. While in humans the utilization of these cells is still considered experimental (except in bone marrow transplants for the treatment of hematological diseases and skin regeneration [5–7]), in veterinary medicine the number of animals already treated provides a substantial basis for assessing the effectiveness of cell therapy in the treatment of a large number of diseases [8, 9]. However, in general, the therapeutic issues involving the use of stem cells to regenerate tissue still have not been fully understood.

Almost all animal tissues may be repaired or regenerated by the direct action of stem cells [10], which presents a high potential for multiplication and differentiation [11]. In this way, a huge effort has been made for understanding the mechanisms by which adult stem cells are able to perform the function of tissue renewal, as well as the conditions that support these processes in organisms affected by diseases. Progressively, adult stem cells from different sources, mainly bone marrow and adipose tissue, have been used for treatment of animal diseases around the world [12, 13]. In this context, the mesenchymal stem cells (MSC), derived from the mesoderm and neuroectoderm [14] and distributed in all vascularized adult tissues (such as adipose tissue, skin, heart, brain, vessels, bones, and cartilage [15]), present an important regenerative capacity.

The MSC have the natural ability for multipotency, being capable of generating new cells of tissues derived from this germ layer. These cells, by action of growth factors and hormones, acquire morphophysiological aspects pertinent to their location within the body (the* niche*) [16]. As far as stem cells drive the different lines within the tissues, these new cells have limited proliferative capacity, being named* progenitor cells*, which in turn are able to differentiate into fewer cell types when compared to the MSC. The MSC and progenitor cells are the main cell types responsible for tissue repair and maintenance in situations of malfunction or injury [17, 18], responding to specific stimuli [19]. Because of this characteristic, these cells have been isolated and used in cell therapy protocols around the world.

In the veterinary field, the MSC, isolated from bone marrow or adipose tissue, through minimal manipulation, have been applied for treating tendon, ligament injuries, and joint diseases, with significant clinical relevance in horses and dogs in orthopedic conditions [9]. However, controlled and well-designed studies of the basic biologic characteristics and properties of these cells are still necessary [20]. In this context, this review will focus on the approaches in the field of veterinary diseases that may be treated through the use of stem cells, emphasizing the protocols that have been more often used in companion, working, and farm animals, and the cell types applied. Besides, the molecular and physiological mechanisms of the regenerative process modulated by MSC and how the benefits obtained in veterinary treatments may be translated to human health will be discussed.

## 2. What Is the Mesenchymal Stem Cell and Why Do We Use It?

### 2.1. MSC Potential for Proliferation, Differentiation, and Tissue Regeneration

**Figure 1:**
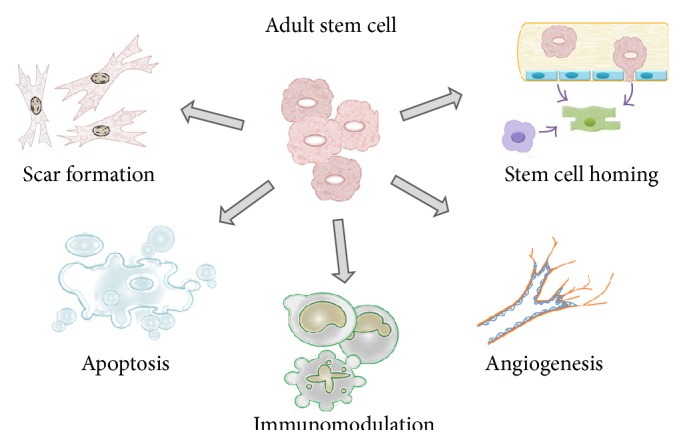
The influence of MSC on the niche. According to the signaling factors that the MSC are exposed, different decisions may be taken, where the main ones are those that are involved in proliferation and cellular differentiation. The MSC capture and send molecular signals, which change the niche, either by modulating the immune system as providing mechanisms for tissue repair effectors, involving since activation of cell homing, cell apoptosis, induction of the formation of new blood vessels, and the healing process.

Despite its potential for plasticity (ability to differentiation) and although cell lines can be successfully derived* in vitro* [21], the use of embryonic stem cells in medicine is still controversial: in humans, mainly because there are ethical and religious issues; in other mammals, because this population is not yet thoroughly exploited; and in both cases there is the eminent possibility of teratoma formation* in vivo* [22] and evidence of genome instability caused by* in vitro* passaging [23]. In this context, the embryonic stem cells have been used in preference to deriving lineages* in vitro*, and the induction of pluripotency in adult stem cells has been explored, which resulted in the* induced-pluripotent stem cell* (iPS), which in turn is emerging as the focus of many studies and some cell therapy protocols [24, 25]. On the other hand, as the embryonic cells differentiate the tissues derived from the three germ layers* in vivo*, the plasticity and potency ability of the generated cell lines is diminished inside the layers. In this way, the mesoderm, germ layer that develops to the muscles, circulatory system, urinary and reproductive tracts, bones and cartilage, connective tissue, and bone marrow, has a pool of self-stem cells, which are named* mesenchymal stem cells* (MSC, as seen above). Conceptually, MSC are cells that display self-renewal capacity (in this case, performing asymmetric cell division, generating undifferentiated cells, and keeping the “stem” capacity) and have potential for differentiation into other cell types [19]. These will be influenced by the niche, the environment necessary for adult stem cells receiving “information,” arising from processes of cell signaling (autocrine, paracrine, and endocrine or intracellular), to activate their mechanisms of cell proliferation and differentiation. This information is prevenient from cell-cell interactions between stem cells, as well as interactions between stem cells and neighboring differentiated cells, interactions between stem cells and adhesion molecules, extracellular matrix components, oxygen tension, growth factors, cytokines, and the physicochemical nature of the environment [16, 26]. The signals emitted by the niche can lead the MSC to assume different “behaviors” [27], depending on the necessity (Figure 1).

Adult stem cells, mainly the MSC compartment, are present in all tissues and organs, and its primary function is the replacement of dead cells in the physiological cell renewal processes [28]. In addition, they may also substitute dead cells in pathological situations, such as ischemia, inflammation, or trauma. Adult stem cells are sources for damaged tissue repair, as they are ready to mobilize in response to injury signaling or pathological conditions [29]. The way by which niche is able to orchestrate stem cells to understand and respond to signals is quite challenging from the point of view of molecular mechanisms, development, and aging [30]. Besides, one of the ways to reproduce these signals is based on the release of a class of small molecules able to bind to specific receptors on the surface of stem cells and immune system's cells, called chemokines. In this way, as soon as the contact occurs between the ligand and the receptor and the higher the number of ligands is, the higher the activation of receptors will be and the greater the favoritism to a positive allosteric gradient is [31]. Thus, the cell assumes signaling pathways that trigger, in addition to migration, a focused cellular response, leading to proliferation or cell differentiation. The more favorable the allosteric gradient is, the better and faster the “decision” by the stem cell will be, which is receiving different signals to perform different functions. Therefore, regarding the need of cell replacement, the type which best responds to the chemotactic signal is the MSC [32]. The perception of this pattern is related to the activation of a specific cellular signaling axis and its response (migration, proliferation, and differentiation), a process named* cell homing*, the essential mechanism for the effectiveness of cell therapy.

Once an injury occurs, cell homing is activated in order to promote the tissue repair. The injured tissue (inflammation), once in an ischemic state, through deprivation of nutrients, generates a hypoxia profile that induces the activation of the hypoxia-inducible factor-1 (HIF-1), which causes the release of cytokines, such as the stromal-derived factor-1 (SDF-1) [33] and the vascular endothelial growth factor (VEGF). These cytokines are locally recognized by the cells and in the blood vessels they help them to cross the tissue layer (transmigration) in order to model the extracellular matrix and activate the necessary cell differentiation. At the same time, the lesions activate the immune response and the consequent release of reactive oxygen species [34], which influences the cell homing. In this context, the injured tissue may comprise some thousands of cells. Further, the lesions may be caused by disease, trauma, excessive physical training [35], undergoing the influence of hypoxia, physical or chemical agents (with or without therapeutic purposes), infectious agents, immune reactions, disease or genetic disorders, and/or nutritional disorders [36, 37]. Thus, once the injury occurs the homing mechanism is readily activated, involving the entire immune system, and there occurs intense mobilization of adult stem cells, potentially the hematopoietic stem cells (HSC), MSC, and other resident tissue progenitor cells (from vascular endothelium, muscles, heart, liver, kidneys, bones, epithelium, etc.) [10, 29]. Consequently, it is important to understand that adult stem cells, particularly tissue-specific isolated or, in contrast, used to regenerate a particular tissue, should be able to recognize the homing orientation to be directed to the lesion site and perform the appropriate repair. Among the MSC sources, the bone marrow and adipose tissue [38] are still the most widely used in therapeutics, although new potential tissues (including synthetic) are being explored.

### 2.2. Procurement and Cultivation of MSC in the Laboratory

Although more conventionally isolated from the bone marrow, MSC are more abundant in adipose tissue. Identified in the vascular fraction (SVF) of this tissue, MSC may be cultivated producing purified populations with high potential of differentiation and secretion of bioactive factors [39]. The combination of these properties has driven the adipose tissue derived MSC (ADSC) research in the last decade, with particular interest in cell therapy and tissue engineering. Recently, the criteria for definition of this cell type have been established by the International Society for Cellular Therapy [40], being similar to those used for bone marrow MSC.

In order to be applied in human or veterinary clinic, stem cells should be “easily” obtained and must be minimally handled, in contemplation to avoid the risk of any kind of contamination. Commonly, bone marrow is collected by aspiration biopsy puncture in the femur of dogs and cats and in the sternum of horses [41]. The samples collected are sent to the laboratory and in appropriated conditions are centrifuged for separation of mononuclear layer. The mononuclear fraction can be prepared in syringes for direct application in the patient or submitted to cultivation for MSC establishment. The cultivation of these cells follows standard cell culture conditions, that is, incubation in a commercial culture medium (usually supplemented with a serum of animal origin to provide a source of growth factors and amino acids), antibiotics, and strictly controlled temperature and humidity (usually using 37°C and 5% CO_2_). The cells remain under these conditions in special bottles where, through subsequent proliferation and population doubling, they expand covering the area of cultivation. Thus, the MSC are characterized by behaving cellular monolayer and display fibroblast aspect in phase-contrast microscope [42]. At each time that the expansion is about to fill the entire area, cells are enzymatically collected through trypsin or collagenase and recultivated. This process is named* cell passage* and is crucial to remove other cell types from culture and indirectly measure the senescence. Usually, after the 4th passage, which can take some weeks, depending on the culture conditions and of the donor organism (healthy or not, young or old, etc.) [43], the MSC are ready for clinical use. Although the healthy stem cell reaches senescence only after more than 10 passages in culture, the prolonged cultivation may interfere with its ability to differentiation [44]. Additionally, MSC are also able to go through the process of freeze/thaw (cryopreservation) while keeping the stem features [45].

The adipose tissue is usually collected aseptically from the inguinal region in dogs and cats and the dorsal surface of the gluteus maximus in horses and then forwarded to the laboratory. Our research group, in order to experimentally analyze the angiogenic response to induction of ischemia by myocardial infarction in minipigs, used the submental fat [46]. In this tissue, stem cells are embedded in extracellular matrix of SVF. The isolation of stem cells derived from adipose tissue is a process based initially on mechanical fragmentation and, subsequently, chemically through digestive enzymes [47, 48]. Mechanical fragmentation is performed using sterile surgical material such as tweezers, scalpel blades, and scissors. The next step, chemical fragmentation, is made by using enzymes such as collagenase, which digest the tissue releasing cells. The resulting mixture, comprised of adipose tissue and enzyme solution, is incubated in physiological temperature to enable enzyme activity and, after the incubation period, the already fragmenting tissue is centrifuged. The final product is the SVF, which precipitates at the bottom of the tube.

The SVF may be prepared in syringes and injected in patients or placed in culture for obtaining, through* in vitro* proliferation, the ADSC population. The option to expand* in vitro* allows, in a few weeks, the emergence of a more homogeneous population of stem cells derived from adipose tissue. In the same way as for MSC, for application in patients, ADSC are collected and placed in syringes with saline solution, being ready for use [49]. Because of its easy collection, abundance of stem cells, and facility of expansion, adipose tissue has been used with much greater frequency as a source of cells for veterinary cell based therapy [38, 49–51].

As for the choice between bone marrow or adipose tissue stem cells, mononuclear fraction, or SVF or after expansion in culture (bone marrow MSC or ADSC, resp.), the decision concerning the optimal condition for its use depends on a number of considerations. The best application will consider the type of pathology, age, and medical characteristics of the patient and the emergency of treatment (considering cell homing, these conditions would be the same for humans or other mammals). The freshly collected fraction, in addition to MSC, contains several other types of cells, as well as signaling molecules like cytokines and growth factors [41]. This whole collection of cellular components and biomolecules has the role in accelerating the regeneration process. Considerations regarding these mechanisms, by which the stem cell operates, and how to modulate them might be extremely beneficial from a therapeutic point of view.

### 2.3. Understanding the Paracrine Effects

The MSC are cells that secrete factors and cytokines that exert influences on tissue repair. Accordingly, many of these molecules are recognized as paracrine agents, which have the function to supply the need of a group of adjacent cells by their influence on activation (or inactivation) of receptors and intracellular pathways without compromising other cells of the body. Under the condition of hypoxia, once the homing is activated, MSC can release, in addition to SDF-1 and VEGF, fibroblast growth factors (FGF) 2 and 7, hepatocyte growth factor (HGF), angiopoietin-1, transforming growth factor-beta (TGF-*β*), matrix metalloproteinase-9 (MMP-9), tumor necrosis factor-alpha (TNF-*α*) and interleukin-1 (IL-1) and interleukin-6 (IL-6), and others [52–54].

After activation of the SDF-1 by injury, performing or not the cell therapy, there is recruitment of adult stem cells (bone marrow or tissue residents). The stronger the signal (usually in the form of a positive allosteric gradient), the more efficient the cellular response. For this to occur, it is essential that the cell is able to express the surface receptors for the signs and, once recognizing the “command to repair,” activates transduction pathways for chemotaxis. Thus, the SDF-1 binds to the specific membrane receptor CXCR4 and activates the MAPK, Akt, PKC, PI3K, and NF*κ*B pathways [55]. These signaling molecules cause activation of cellular proliferation, cytoskeletal reorganization, and induction of other cytokines, particularly interleukins.

The activation of the angiogenic process is crucial for the formation of new blood vessels, improving the nutrition of injured tissues and consequently contributing to the recovering of ischemic areas. Whether after hypoxia or establishment of the inflammatory process, both VEGF and angiopoietins (responsible for the vessel maturation) have their expression induced [56] and activate endothelial progenitor cells that will constitute new vessels. A study in pigs with induced proctitis showed that repeatedly injected MSC were able to modulate the expression of VEGF and its receptor, in addition to angiopoietins and FGF 2 [57]. Further, cell therapy protocols targeting the induction of angiogenesis are well studied for muscle tissues and limb ischemia and, mainly, in cardiovascular diseases. However, it is very important that the use of therapies able to induce angiogenesis observes the presence of neoplasia, which is very common in dogs and cats of advanced age [58]. The process of tissue nutrition is also very important as regards the modulation of extracellular matrix, which is the basis of tissue and cellular organization.

To understand the relationship between injuries of skin, muscle, bone, and cartilage and the repair potential offered by MSC, it is necessary to have a deeper insight about the functions of the extracellular matrix. The extracellular matrix, whose main function is tissue support (through the “fixing” of cells in tissues and to each other), is organized with fibrous and fluid elements. The central fluid element is composed by the glycosaminoglycans, which by its hygroscopic capacity, form glycoconjugates that provide resistance to compression forces, acting as lubricant for the joints and tendons. The glycosaminoglycan matrices regulate the passage of molecules through the extracellular space, participating in the maintenance of chemotactic gradient blocking, stimulating or guiding the migration and cell dispersion, and, in this way, influencing the stem cells' repair function [59]. These molecules are the basis of the cartilage. On the other hand, the fibrous elements are structural proteins, such as collagen, which forms flexible and inelastic fibers with great tensile strength. The extracellular matrix is regulated by a specific family of proteins, the* matrix metalloproteinases* (MMPs).

The MMPs cause degradation of protein components of the extracellular matrix and, with their specific inhibitors (TIMPs), these molecules are able to modulate the stem cells homing. Bhoopathi et al. showed that the inhibition of MMP-2 was able to inactivate the tropism of MSC towards tumor, for its influence on the SDF-1/CXCR4 axis, in mice presenting medulloblastoma, in addition of induction of cytokine profile changes [60]. Therefore, it should be noted that the inflammatory process is extremely active on the structure and function of the extracellular matrix regarding, for example, the changes in cartilage and alterations on its biomechanics, as those occur in cases of osteoarthritis [61]. Besides, MSC have great ability to cause healing processes for their influence on inflammatory proteins and, consequently, on extracellular matrix-forming molecules, as collagen [62] and fibronectin [63]. On this basis, the combination of MMPs and MSC emerges as a “double-edged sword”: the first recruits the second that modulates the first, and together they may act on repair of tendon injury and osteoarthritis. In this context, the ability of the MSC to influence the environmental repair is intimately related to its immunomodulatory role.

### 2.4. MSC Immunomodulation Potential

The immunomodulation function of MSC is related to its ability to repair, performed throughout the life of the organism. This characteristic also results in tolerance by the recipient's immune system allowing the use of genetically different donor cells (allogeneic) and is greatly appreciated in cell transplantation protocols [64]. The immunomodulatory capacity of MSC seems to be related to its interaction with T-CD4 and T-CD8 cells and their proinflammatory mediators. This class of stem cell reverses the inflammatory sign through the downregulation of the secreted mediators and activating anti-inflammatory cytokines [65, 66]. In fact, T-cells are directly involved in graft* versus* host disease, as demonstrated by Polchert et al., in response to IFN-*γ*, which activates TGF-*β* but had no effect on inducing IL-10; MSC increase suppression and limit Th1 responses [64]. Also, Chiesa et al. showed that the MSC were able to prevent the presentation of antigens by dendritic cells to lymphocytes, as well as their migration to lymph nodes (activation location) [67].* In vivo* administrated MSC generated a significant downregulation of CCR7 and CD49d*β*1, two molecules involved in the dendritic cells homing to lymphoid organs.

The ability to inhibit the proliferation of stimulated T cells* in vitro* has been well described for MSC in a large number of nonhuman species. In dogs, MSC were able to increase IL-6 and TGF-*β* and decrease TNF-*α* [68]. In chickens, the inhibition of T-lymphocytes by MSC was correlated to nitric oxide production [69]. A guinea-pig model for acute colitis showed that bone marrow MSC, as compared to adipose tissue stem cells, appeared more effective in the attenuation of plexitis, reduction in choline acetyltransferase immunoreactivity, and consequent lymphocyte infiltration on the level of the myenteric ganglia [70]. Carrade et al. pointed out that in horses, similar to what happens in humans and rodents, once stimulated, MSC of all tissue types decreased lymphocyte proliferation, increased prostaglandin E2 and IL-6 secretion, and decreased production of TNF-*α* and IFN-*γ* [71], as already seen previously. Similar results and mechanisms may be observed in other species [66]. Haddad and Saldanha-Araujo also discussed the main mechanisms through which MSC immunosuppress T-cells and their response, which focuses on cell-cell contact, secretion of soluble factors, and regulatory T-cell generation [72]. According to the authors, surface markers and toll-like receptors of MSC (isolated from different sources) and their induced-cytokines counterparts may influence the inflammatory microenvironment (niche) and also the immunosuppression process.

All the information concerning the best organic response must be gathered in time to cell therapy application, as well as which cell type, the delivery route, and the number of applications. Other than that, the patient health state must be well evaluated in order for the stem cell homing to be effective.

## 3. Cell Therapy Applications in Veterinary Medicine

In veterinary medicine, since the early 2000s, cell therapy is a clinical reality, where the first applications were intended for the treatment of tendon injuries in horses [73]. Several companies worldwide offer the service of isolation of bone marrow mononuclear cells, bone marrow MSC, or ADSC for treatment primarily of companion animals [8] and horses [13]. Although there is still need for randomized and controlled clinical studies, thousands of animals already treated in the world enabled an evaluation of the effectiveness of this therapeutic procedure. Mononuclear fraction and MSC have been employed mainly in veterinary medicine for treating tendon and ligament injuries and joint diseases in horses and other species, with minimal manipulation of cells [9]. As will be discussed below, studies and clinical trials show that autologous bone marrow cells have an important therapeutic potential and present clinical benefits in horses and dogs in orthopedic conditions. Furthermore, the number of studies using MSC or ADSC has grown in treatment protocols, with or without the use of biomaterials as scaffolds [50].

### 3.1. Bench to Bedside

**Figure 2:**
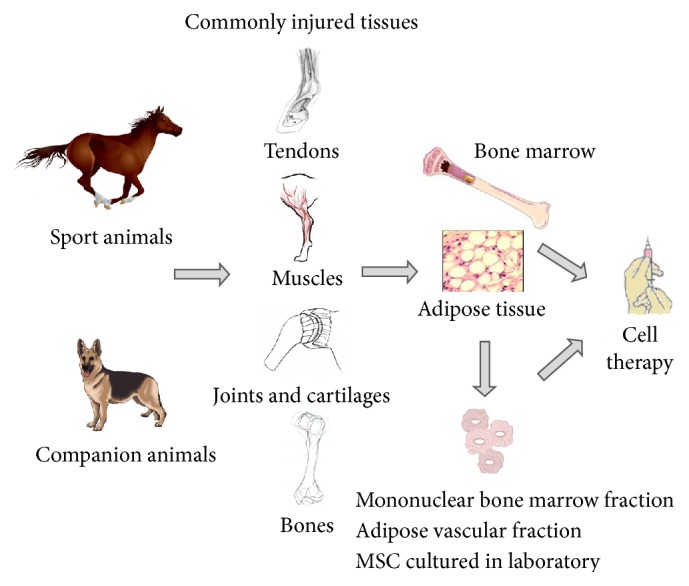
Cell therapy applications in veterinary medicine. Companion animals (pets), due to genetic factors, degenerative processes, and inadequate diet, and animals used in sports, such as running and jumping, are subject to several kinds of injuries, which may affect primarily the muscle tissue, causing pain, cartilage wear in joints and spinal disks, tendonitis, fractures, and bone degeneration. Clinical applications and protocols are based on the use of adult stem cells, isolated from fresh bone marrow or adipose tissue, or expanded from these tissues in laboratory. The derived cells, mesenchymal stem cell (MSC), have a high therapeutic capacity.

Cell therapy in dogs, cats, and horses depends on the veterinarian's contact with the laboratory that isolates, cultivates, and prepares cells for application. At that point, veterinarians and laboratory professionals must decide together what the best form of treatment is: fraction directly collected from the patient (bone marrow mononuclear fraction or SVF) or cultured cells (MSC or ADSC, resp.), autologous or allogenic donor. After the decision about the cell type, the professionals organize the collection and sending of the cells. If the option is to use the mononuclear fraction or SVF, the veterinarian receives a kit from the lab, makes the aseptic collection of bone marrow or fatty tissue, and sends the material back to the lab. Once received, the isolation of cells takes 2-3 hours. Due to viability and cell behavior, for horses (e.g.) it is recommended that the cells are applied within 24 hours after preparation and the use of large bored needles is also recommended [74]. Considering that the number of isolated cells is directly related to the size (mass) and quality of the tissue (either adipose tissue or bone marrow), the quantity applied is also variable, showing an absence of specific standardization to each species or disease. In accordance with the protocols described below, some injected volumes will be presented.  Figure 2 outlines some cell types and applications in the veterinary clinic.

### 3.2. Animals in Sport

Although there are a large number of sports involving animals, such as hunting, fighting, and bullfighting, it is in the racing modalities that cellular therapy protocols are more commonly applied. That is because this modality is the one that causes most injury to tendons, joints, cartilage, and bones in horses. For this veterinary class, jumping is also considered as an activity that causes those kinds of injuries. Beyond the problem of animal health, the injuries, in turn, generate considerable economic problems, since horses are animals that involve significant costs to their physical training and competitiveness. Due to the easy collection and effectiveness, the largest number of protocols aimed at administration of cell therapy for horses was based on the use of bone marrow mononuclear fraction [75, 76]. More recently, probably through the search of strategies that might ensure even better results, other cell types began to be investigated.

Initially, after exhausting training, even if there is no cartilage or bone injury, the animal may be affected by muscle aches, which hamper its locomotion and can exacerbate inflammatory processes [34]. Considering the musculoskeletal injuries, Torricelli et al. showed that racing horses who were given 1–4 × 10^6^ mononuclear bone marrow cells, combined with autologous platelet-rich plasma, applied directly onto the lesion (located by ultrasonography), showed muscle regeneration after 12 months of follow-up [13]. Thirty animals were submitted to the cell therapy, where 28 were able to return to racing, showing that the protocol has generated noticeable improvements. Further, since MSC have the facilitated potential to differentiate in cell types of these tissues, they have been recently pointed out as promising candidates for cell therapy and regenerative medicine to the equine patient undergoing musculoskeletal injuries [38]. Soft tissue injuries of the musculoskeletal system are common in humans and animals, especially athletes, and lead to considerable morbidity in both patient populations. Fortunately, these lesions appear as good targets for the effectiveness of cellular therapy, mainly with MSC.

Tendons are fibrous structures with function of connective tissue, with intertwined collagen fibers, and are attached to bones by annular ligaments. The recovery of damaged ligaments and tendons occurs with large influx of cells and formation of new fibrous tissue, but with lower biomechanical property, leading to performance fade, as well as increased risk of new injury. It was also pointed out that* tendon-derived stem cells*, able to differentiate into other cell types, such as muscle or fat, present in the injury could be liable to aggravate the pathological situation and cause chronic tendinopathy [77]. Horses and dogs are among the most clinically affected animals, with naturally occurring tendinopathies with histopathologic similarities to those observed in humans when analyzed by MRI and ultrasonography [8]. The repetitive stress injuries of the digital flexor tendon are common in racing and jumping horses [73].

Due to the high presence of collagen, the injury promptly induces activation of the homing mechanism, which allows the tendon repair by stem cells injection. Moreover, there are an increasing number of experimental protocols describing improved outcome after the use of a combination of stem cells and integrated genes to stimulate the tendon regenerative process [78]. The principal growth factors evaluated include bone morphogenetic factor (BMF), platelet-derived growth factor (PDGF), FGF, VEGF, and insulin-like growth factor 1 (IGF-1) [78, 79]. Tetta et al. observed that exosomes (extracellular vesicles involved in cell-cell communication) released by the MSC could potentially secrete anti-inflammatory factors, which would be very efficient to the tendinous repair [80]. Filomeno et al. showed that adult stem cells treated with platelet-rich plasma could also be promising for veterinary and human trials [77]. In this context, horses submitted to the application of 1 × 10^7^ ADSC combined with autologous platelet concentrates were able to prevent tendonitis progression, in a follow-up of 16 weeks, showing greater organization of collagen fibers, and decreasing in the inflammatory infiltrate when these animals were compared to the control group (PBS-treated) [73].

In the case of soft tissue regeneration, as muscle and tendons, it is also important to note that, in addition to the fiber organization, the formation of new vessels is quite important for their nutrition. Thus, therapy with stem cells must be able to activate local homing to induce the production of angiogenic factors such as VEGF and angiopoietins. In fact, trophic factors secreted by equine MSC were able to induce angiogenesis* in vitro* through activation of VEGF [79]. However, intrinsically, angiogenesis may elicit more inflammation because it makes the tissue more permissive to the migration of cells of the immune system. Therefore, even if shown as active agents in development of degenerative tendinopathy, the tendon-derived stem cells are being studied and were pointed out to proliferate more quickly than tenocytes in culture, and when implanted* in vivo* these cells exhibit the ability to regenerate tendon-like tissue [77]. The authors also discussed that the degenerative process maintenance may be explained by the fact that tendon stem cells might be influenced by early senescence, caused during the large inflammatory state associated with tendinopathy establishment.

Through the use of MSC, derived from bone marrow or fatty tissue, therapeutic protocols are a viable reality, but because tendons of racehorses are a permanent problem new approaches have been studied. In this way and, as a manner to keep the cells at the site of injury and optimize repair, biomaterials have been the subject of much research effort [75]. Fibrin and collagen-based hydrogels have been widely used for tendon repair due to their low antigenicity and immunogenicity and their inherent properties, such as cell recognition signals to promote cell attachment, cell homing, proliferation, differentiation, and consequent stimulus to tissue healing and regeneration [81, 82]. For its effectiveness, the use of stem cell carriers, beyond low invasiveness, assists in targeting* in situ* cell injection and therapeutic maintenance [83].

Sport activities are also able to cause damage in cartilages, especially in joints and other articulations, and veterinary medicine has also been concerned with these injuries [84, 85]. In fact, these structures have a regenerative capacity lower than other tissues. Traumas caused in these cartilages lead to ligament desmitis, a form of lameness that affects performance, and osteoarthritis, a degenerative disease that may cause pain, stiffness, and reduced functionality of the joints. Even though these lesions cause stimulation of stem cells homing, physiologically the mechanism is weakly activated. In this context, it is known that the MSC have high potential for differentiation into osteogenic and chondrogenic cell lines [86]. However, there is discussion about which cell type (from bone marrow or adipose tissue) would prove to be of most advantageous use in veterinary medicine.

Vidal et al. compare the chondrogenic potential of adult equine bone marrow MSC or ADSC* in vitro* [87]. Both cultured cells were induced into chondrogenesis using TGF-*β*3 and BMP-6, where the synthesis of glycosaminoglycan and collagen type II was analyzed. Equine bone marrow MSC showed superior chondrogenic potential when compared with ADSC, indicating that those cells respond in a better way to stimuli provided to the cartilaginous tissue regeneration. The use of allogenic MSC, commercially produced, was also tested for ligament desmitis in a case study [88]. Four weeks after the diagnosis, the patient received an injection of MSC combined with platelet-rich plasma and, after 12 weeks, a second injection, under the same conditions, in order to stimulate healing. After 32 weeks, through monitoring by ultrasound, the animal showed total restoration of fiber alignment and exhibited performance standards related to the initial level, before the development of the disease. Thereby, considering that in veterinary medicine the MSC may be used in allogenic condition (which still is not defined for human patients), this facilitates the process of cell homing, once the cells are commercially available from healthy and, in general, younger individuals (with increased proliferative rate). Broeckx et al., in a clinical study with horses affected by degenerative joint disease, a major cause of reduced athletic function and retirement in equine performers, tested the individual and combined use of MSC and platelet-rich plasma [85]. The cells were isolated from peripheral blood of a 6-year-old animal and MSC cultures were established and tested for their identity. Samples of MSC also were submitted to chondrogenic differentiation* in vitro*; the MSC were also tested for expression of MHC Class II. After 12 weeks it was verified that MSC did not show MHC expression and the combination of protocols (MSC + platelet-rich plasma), with or without chondrogenic induction, was able to reverse the injury, improving functionality and sustainability of damaged joints.

Problems involving injuries of soft tissues, cartilage, and bones, as seen, are fairly common in animals subjected to high-impact sports, especially in horses. However, degenerative processes in these tissues also occur in animals for company, like dogs and cats.

### 3.3. Companion Animals

In recent years, there has been great interest in the use of stem cells as therapy for a variety of diseases in domestic animals, mainly those of company. Although the scientific literature features a marked disparity between the supposed benefits of stem cell therapies and their proven capabilities in human, as defined by rigorously controlled scientific studies, stem cells implanted therapeutically offer innovative treatment perspectives for diseases considered so far without cure in companion animals.

Recently, the morphology and physiology of MSC isolated from bone marrow have been evaluated in different canine breeds (Border Collie, German Shepherd, Labrador, Golden Retriever, and the Hovawart) [89]. Proliferative capacity-based analyses, senescence, cell lines* in vitro* differentiation, and phenotypic characterization showed that the behavior of MSC was quite similar in all races tested, albeit with some particularities (e.g., Border Collie is a breed that has cells with greater capacity for cell division and osteogenic differentiation; Shepherd, Labrador, and Golden had higher percentage of senescent cells; etc.). Thus, regardless of some variations, the MSC were isolated from the bone marrow and established in culture, where all the analyzed breeds were able to be expanded* in vitro*, showing potential for proliferation and differentiation, and thereby becoming eligible for cell therapies.

As seen for the equines, the potential clinical use of adult stem cells aroused commercial interest from several companies, where the purified cells have been even tested in research protocols for treatment of canine bone arthritis. Thus, it is in the orthopedic treatment of dogs that the bone marrow mononuclear cells have been more concerned as therapeutic method [12]. It is understood that these cells are able to recruit other stem cells and the immune system cells, thus inducing homing* in vivo* [90]. The bone marrow cells have high affinity for the injured tissue and, considering its potential in repairing injuries, are able to regenerate damaged structures in the joints, ligaments, menisci, and cross-like lesions of the cartilage, in a process quite similar to those that occur in horses and other animals [91].

Despite the benefits obtained with the use of cells isolated from peripheral blood, depending on the animal state of health and age, the response of these cells can be weaker in relation to other cell types such as the MSC. Nevertheless, older dogs present common problems involving the circulatory system. Hulanicka et al. analyzed the transcriptional profile of nuclear cells isolated from the peripheral blood of dogs with heart failure and found that these cells exhibit altered expression of molecules—potentially inductors of pathological processes [92]. An example was about MMPs, whose erroneous activation can lead to improper extracellular matrix remodeling. On the other hand, the use of bone marrow stem cells and progenitor cells for dogs with heart diseases is promising, due to protocol safety and feasibility of application, which may be done by intracoronary infusion [93].

The use of MSC isolated from adipose tissue has also been very often applied in dogs. Even the SVF or ADSC may be easily obtained from subcutaneous adipose tissue through liposuction procedure, showing good proliferative potential and plasticity [45]. Nevertheless, some protocols have already been performed applying these cells. Ryu et al. evaluated the implantation of 1 × 10^6^ allogenic ADSC in dogs with acute spinal cord injury [94]. Magnetic resonance imaging and histopathology to mature neural cells revealed that the group treated with cells improved the nerve conduction velocity, the somatosensory potential, and the neurological function. Allogenic ADSC were also used to treat canine patients with hip dysplasia [12]. In this study, the SVF was collected from the inguinal region and applied in concentrations between 2 and 5 × 10^6^ cells in autologous way (*n* = 9); ADSC were established from SVF for 4 weeks and used as 2 to 8 × 10^5^ cells in allogenic condition (*n* = 4); both cell types were injected in acupuncture points near the affected joints. Cell therapy was safe and successfully assured improvement after 30 days in 70% for SVF and 50% for ADSC.

Dogs and cats may present bone problems, either hereditary or caused by some malformation or inadequate diet [94]. The main problems are the rickets, osteomalacia, osteoporosis, osteofibrosis, and other skeleton malformations. Considering the bone repair and lengthening (*osteogenesis*), the MSC are also a good therapeutic option. Interestingly, in addition to its intrinsic activity, this cell type may also serve as gene carriers (*ex vivo* gene therapy) of pharmacological agents that, in turn, can assist in the osteogenic process [95]. Besides, the authors point examples of factors that help to repair bone cell homing as FGF, BMP, and the Wnt/*β*-catenin and Notch signaling pathways. A study also tested the transplantation of bone marrow MSC and plasma-rich platelet to distraction osteogenesis in dogs [9]. Cells, 1 × 10^7^, were directly injected into tibia callus and after 3 months, radiography, computerized tomography, and histology evidenced that the treated group showed significant and appropriate tibia lengthening. In this way, for bone repair, the bone marrow MSC established in laboratory appears to be more attractive in relation to the fresh bone marrow fraction.

Even in view of the use of multipotent stem cells in regenerative veterinary medicine, Park et al. isolated and used MSC isolated from amniotic membrane of dogs [96]. Authors performed differentiation lineage protocols and verified the multipotent ability, under the appropriate culture conditions, showing that these cells may be a rich source of allogenic stem cells in dogs. Based on this idea, Horie et al. tested the implantation of synovial-derived MSC to treat rabbit knees with partial meniscectomy [97]. After 24 weeks, the implanted cells adhered to meniscal defects and activated the tissue regeneration, where they differentiated into type-I and type-II collagen-expressing cells. Bone diseases such as osteoarthritis and problems in cartilage and joints also affect domestic cats. Analyzing and considering the information regarding age and pathological conditions, most adult stem cell protocols, as those used in dogs, may also be extrapolated to cats. Nevertheless, some of these diseases are also found in farm animals, destined for slaughter.

### 3.4. Farm Animals for Slaughter

Nowadays, one of the major concerns is obtaining agricultural and cattle inputs for the nutrition of world population. In this way, there is a great concern for cattle destined for slaughter, where the husbandry environment (confine or pasture) and physical development to obtaining of better meat and reproductive potential should be considered. Although stem cells are still little explored in this field, there are already some caprine models for treating cartilage injuries and cell types, as the iPS, being studied.

The cattle creation, where it aims to develop fat to obtain tender meat, sometimes by their highest weight, may induce cartilage and bone problems. Further, especially when in grazing, this may influence their locomotion. Nam et al. proposed the identification and establishment of MSC for repair of caprine chondral injuries [98]. The bone marrow was aspirated from the iliac crests and MSC were established* in vitro*. Histological and immunohistochemical analysis demonstrated hyaline-like cartilage regeneration and glycosaminoglycans synthesis in the transplanted sites of the group that received 1 × 10^7^ MSC compared to control or bone marrow-treated goats. The intra-articular injection of MSC may provide superior cartilage repair outcomes and that could be extrapolated to other farm species.

The bovine mastitis, an inflammatory disease in the mammary gland, causes a drastic decrease in milk production and economic problems. The disease directly affects the farmer's income by decreasing milk production, reduced milk quality, and reduced animal value, while leading to costs with drugs, risk of culling of animals, and possible death. Besides, bovine mammary epithelial cells and their stem cells are very important in milk production and bioengineering. Thus, Sharma and Jeong demonstrate the possibilities of bovine mammary stem cell therapy, offering significant potential for regeneration of tissues that can potentially replace or repair the diseased gland suggesting differentiation of stem cells isolated into epithelial, myoepithelial, and/or cuboidal/columnar cells decreasing risks after reinjection [99]. The authors also believe that the iPS cells could be used in this kind of approach.

Induced pluripotent stem (iPS) cells are defined as differentiated cells that have been experimentally reprogrammed to pluripotent cells, to achieve an embryonic stem cell-like state. In general, these cells are submitted to retroviral transduction for a core of reprogramming factors as* Oct4*,* Sox2*,* Klf4*, and* c-Myc* genes. This cellular reprogramming is quite appreciated in terms of conditioning farm animals to test novel cell therapies, implement pharmaceutical and regenerative studies, and conduct experiments for fertility restoration. Both MSC and fibroblasts may be used for these purposes and there are studies already for the obtaining of iPS to buffalo, cattle, goats, pigs, sheep, and other farm animals [100]. In this way, a study was also conducted to assess the effect of supplementation of different growth factors in embryonic stem cell culture of buffalo [101]. Authors showed efficiency of blastomere attachment, formation of embryonic stem cell-like colonies, and their propagation* in vitro* and characterization, concluding that these buffalo cells presented high potential of pluripotency. In addition, the stem cell manipulation is already a reality with regard to improving reproductive health. However, the use of stem cells to increase the clonogenic potential still lacks many studies. Among these, it must be considered how basic issues such as epigenetics, which causes blockage in the transcription of genes, can influence the success of the artificial cloning of domestic animals [102].

## 4. Frontiers of Stem Cells and Regeneration in Veterinary Science and Translation to Human Health

The use of cell therapy in veterinary medicine is now a reality. Many clinics are now making use of stem cells injection, autologous or allogenic, fresh or cultured in the laboratory, for the treatment of diverse veterinary diseases. The available literature supports that the process is safe and brings considerable benefits to animal health. The knowledge about how adult stem cells interact with niche molecular signals in order to activate cell homing also has increasingly been evidenced to determine the mechanisms by which cells are competent or not in repairing tissues. Thus, beyond the direct clinical application, many animal models have been addressed and/or tested for these purposes. These findings contribute primordially to the translation to the human clinic, where most of the protocols developed were geared towards the cardiovascular or hematologic area and often did not provide the expected benefits.

Among the most widely used cellular types, an important highlight is given to the MSC. These cells are able to modulate the immune system, activating homing factors and more favorably allowing cells to access the site of injury, benefiting the tissue repair. The different veterinary protocols using MSC derived from bone marrow or adipose tissue clearly point out that this cell, even with required cultivation and manipulation in laboratory, might be much functional if applied in human clinic. On the other hand, the possibility of use of iPS in order to generate cells with pluripotency capacity may also bring great advantage to veterinary science [103]. Nonetheless, caution must be kept in early clinical translation with this cell type, especially regarding safety issues [104]. Finally, special attention should be given to the patient's health: the more compromised it is, the more difficult the promotion of stem cell homing activation and subsequent repair will be. In this way, many efforts are still needed for the determination of mechanisms that promote the cell homing for the best benefit of using cell therapy in veterinary or human medicine.

## 5. Conclusion

In conclusion, this review showed that the cell therapy is a safe approach, not too expensive or laborious, and that can be applied to several species of mammals, as they share their morphological tissue base. Among the stem cells used in procedures, those with the best chance of therapeutic success are the MSC (isolated from bone marrow or adipose tissue), due to their ability to promote tissue repair, activation of paracrine factors, immunomodulation, and perception of the cell homing signaling. Thus, these cells have been more often applied to companion and competition animals to treat bone diseases (such as osteoarthritis), tendons and cartilage, muscles, and other tissues, either caused by genetic origin or developed by physical activity, inadequate diet, and so forth. The applicability of stem cells as therapy may also be exploited in other veterinary groups such as farm animals.
